# 634. Implementation of a medical education intervention to improve acceptance of HIV-positive donors for transplant at a tertiary care hospital

**DOI:** 10.1093/ofid/ofac492.686

**Published:** 2022-12-15

**Authors:** Neeraja Swaminathan, Vagish Hemmige, Jonathan Mamber Czeresnia, Victoria Muggia, Yorg Azzi, Enver Akalin, Harith Raees, Haider Al Anssari

**Affiliations:** Montefiore Medical Center, NY, New York; Montefiore Medical Center, NY, New York; Montefiore Medical Center/Albert Einstein College of Medicine, Bronx, New York; Montefiore, NY, New York; Montefiore Medical Center, NY, New York; Montefiore, NY, New York; Montefiore Medical Center/Albert Einstein College Of Medicine, New York, New York; Montefiore Medical Center/Albert Einstein Medical College, New York, New York

## Abstract

**Background:**

The HIV Organ Policy Equity Act (HOPE Act) was enacted in the US on November 21, 2013. HIV patients have a higher waitlist mortality and decreased access to transplant compared to HIV negative controls. The HIV Organ Policy Equity Act (HOPE Act) was a major step towards increasing the donor pool, but utilization of organs through the act has been less than initially anticipated.. Our own institution performed only one HIV D+/R+ transplant in the first four years of the trial despite a significant number of HIV+ patients on the waitlist.

**Table:**
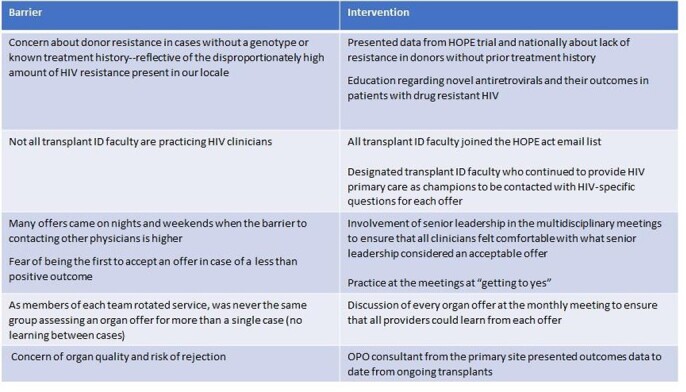
Figure summarizing perceived barriers and targeted interventions.

Rate of HOPE transplants impacted positively by intervention

**Figure:**
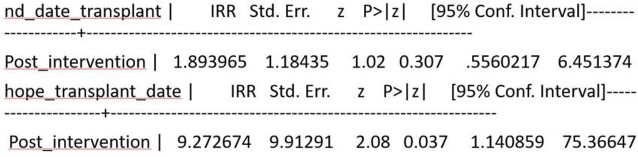
Rate of HOPE transplants impacted positively by intervention

**Methods:**

Monthly multidisciplinary meetings were set up. The agenda included discussing all offers, risks/benefits in a standardized format. Didactic sessions were conducted to address identified barriers to accepting HIV+ organs (Figure 1). HOPE consultants from experienced larger-volume centers were invited as guests.

**Results:**

In the 15 months since this intervention was implemented, there have been 7 HIV D+/R+ transplants including a heart-kidney transplant. While the overall rate of transplant didn't change in a statistically significant way pre and post intervention for patients the rate of HOPE transplants increased significantly (Figure 2).

**Conclusion:**

Making organs from HIV-positive donors available for donation does not mean they will be used. Intensive provider education can improve organ acceptance rates and help fulfill the promise of the HOPE act.

**Disclosures:**

**Vagish Hemmige, MD**, Merck: Grant/Research Support.

